# Hostile‐helpless states of mind: A scoping review of risk factors, correlates, and consequences

**DOI:** 10.1002/imhj.21994

**Published:** 2022-05-31

**Authors:** Jessica Turgeon, Tristan Milot, Diane St‐Laurent, Karine Dubois‐Comtois

**Affiliations:** ^1^ Department of Psychology Université du Québec à Trois‐Rivières Trois‐Rivières Canada; ^2^ Department of Psychoeducation Université du Québec à Trois‐Rivières Trois‐Rivières Canada; ^3^ Centre d’études interdisciplinaires sur le développement de l'enfant et la famille (CEIDEF) Trois‐Rivières Canada; ^4^ Centre de Recherche Universitaire sur les Jeunes et les Familles (CRUJeF) Trois‐Rivières Canada; ^5^ Groupe de recherche et d'intervention auprès des enfants vulnérables et négligés (GRIN) Trois‐Rivières Canada; ^6^ CIUSSS du Nord‐de‐l'Île‐de‐Montréal Trois‐Rivières Canada

**Keywords:** attachment disorganization, Hostile‐Helpless states of mind, adult attachment interview, intergenerational transmission, scoping review, desorganización de la afectividad, estados mentales Hostiles‐Sin Ayuda, Entrevista de la Afectividad Adulta, transmisión intergeneracional, revisión de alcance comprensivo, Mots clés: désorganisation de l'attachement, états d'esprit Hostile‐Impuissant, Entretien d'Attachement Adulte, transmission intergénérationnelle, passage en revue, Bindungsdesorganisation, feindselig‐hilflose Gemütszustände, Adult Attachment Interview, intergenerationale Übertragung, Übersichtsarbeit, キーワード: 無秩序な愛着、無力/敵対的な心性Hostile‐Helpless states of mind 、成人愛着面接 (AAI) 、世代間伝達、スコーピングビュー, 关键词:依恋混乱, “敌对无助”心理状态, 成人依恋访谈, 代际传递, 范围综述, الكلمات الرئيسية: عدم تنظيم التعلق ، حالات عقلية معادية وعاجزة ، مقابلة تعلق البالغين ، الانتقال عبر الأجيال ، مراجعة منهجية

## Abstract

Chronic relational trauma can lead to the formation of pervasively unintegrated attachment representations in adulthood, referred to as Hostile‐Helpless (HH) states of mind. Individuals with this type of attachment disorganization evidence conflicting evaluations of caregivers and have difficulty reflecting on their traumatic childhood experiences. This scoping review is the first to systematically integrate the results of all empirical studies on HH states of mind in an effort to highlight the scientific and clinical contributions of the concept and guide future research. Following Arksey and O'Malley's (2005) Methodological Framework, cross‐reference keywords were searched in three databases (PsycArticles, Psychology and Behavioral Sciences Collection, ProQuest). In total, 19 studies met inclusion criteria and were included in the synthesis. Results suggest that prevalence rates of HH states of mind increase as a function of adults' psychosocial risk status. Findings also reveal that the long‐term consequences of early trauma are greater in the presence of a HH state of mind, whereas the absence of a HH state of mind acts as a protective factor against the intergenerational transmission of maladaptation. Finally, results support the discriminant validity of the HH classification against other forms of attachment disorganization in adulthood. Research gaps and future research directions are discussed.

## Resumen

El trauma crónico de la relación puede llevar a la formación de representaciones de afectividad profundamente desintegradas en la edad adulta, conocidas como estados mentales Hostiles‐Sin Ayuda. Los individuos con este tipo de afectividad desorganizada demuestran evaluaciones conflictivas de quienes les cuidan y tienen dificultades para reflexionar acerca de sus experiencias traumáticas de niñez. Este estudio de alcance comprensivo es el primero en integrar sistemáticamente los resultados de todos los estudios empíricos acerca de los estados mentales Hostiles‐Sin Ayuda como un esfuerzo para subrayar las contribuciones científicas y clínicas del concepto y guiar la investigación futura. Siguiendo el Marco de Trabajo Metodológico de Arksey y O'Malley's (2005), se investigaron palabras claves con enlaces de referencia en tres bases de datos (PsycArticles, Colección de Psicología y Ciencias del Comportamiento, ProQuest). En total, 19 estudios reunieron los criterios de inclusión y se les incluyó en la síntesis. Los resultados indican que los puntajes prevalentes de los estados mentales Hostiles‐Sin Ayuda aumentan como una función del riesgo sicosocial de los adultos. Los resultados también revelan que las consecuencias a largo plazo del trauma temprano son mayores en presencia de un estado mental Hostil‐Sin Ayuda, mientras que la ausencia de un estado mental Hostil‐Sin Ayuda actúa como un factor de protección contra la transmisión intergeneracional de mal adaptación. Finalmente, los resultados apoyan la validez discriminante de la clasificación Hostil‐Sin Ayuda contra otras formas de desorganización de la afectividad en la edad adulta. Se discuten los vacíos y el futuro de la investigación.

## Résumé

Le trauma relationnel chronique peut mener à la formation de représentations de l'attachement non intégré omniprésent à l’âge adulte, auquel on fait référence en tant qu’états d'esprit Hostile‐Impuissant. Les individus avec de type de désorganisation d'attachement font état d’évaluations contradictoires des personnes prenant soin d'eux et ont des difficultés à réfléchir à leurs expériences traumatiques durant l'enfance. Ce passage en revue est le premier à systématiquement intégrer les résultats de toutes les études empiriques sur les états d'esprit Hostile‐Impuissant pour essayer de mettre en évidence les contributions scientifiques et cliniques du concept et guider les recherches futures. En suivant la structure méthodologique de Arksey et O'Malley (2005) les mots clés de référence croisée ont fait l'objet d'une recherche dans trois bases de données (PsycArticles, Psychology and Behavioral Sciences Collection, ProQuest). Au total 19 études ont rempli les critères d'inclusion et ont été inclues dans la synthèse. Les résultats suggèrent que les taux de prévalence d’états d'esprit Hostile‐Impuissant augmentent en tant que fonction du statut de risque psychologique des adultes. Les résultats révèlent aussi que les conséquences à long terme du trauma précoce sont plus grandes en présence d'un état d'esprit Hostile‐Impuissant, alors que l'absence d’état d'esprit hostile‐Impuissance agit en tant que facteur protecteur contre la transmission intergénérationnel de mal‐adaptation. Enfin, les résultats soutiennent la validité discriminante de la classification Hostile‐Impuissant par rapport à d'autres formes de désorganisation de l'attachement à l’âge adulte. Les écarts dans les recherches et des directions futures de recherches sont discutés.

## Zusammenfassung

Feindselig‐hilflose Gemütszustände: Ein Überblick über Risikofaktoren, Korrelate und Folgen

Chronische Beziehungstraumata können im Erwachsenenalter zur Bildung von durchgängig unintegrierten Bindungsrepräsentationen führen, die als feindselig‐hilflose Gemütszustände (Hostile‐Helpless states of mind) bezeichnet werden. Personen mit dieser Art von

Bindungsstörung weisen widersprüchliche Bewertungen von Bezugspersonen auf und haben Schwierigkeiten, über ihre traumatischen Kindheitserfahrungen zu reflektieren. Diese Übersichtsarbeit ist die erste, die die Ergebnisse aller empirischen Studien über feindselig‐hilflose Gemütszustände systematisch zusammenfasst, um die wissenschaftlichen und klinischen Beiträge des Konzepts hervorzuheben und richtungsweisend für künftige Forschung zu sein. In Anlehnung an den methodischen Rahmen von Arksey und O'Malley (2005) wurden Querverweis‐Schlüsselwörter in drei Datenbanken (PsycArticles, Psychology and Behavioral Sciences Collection, ProQuest) durchsucht. Insgesamt erfüllten 19 Studien die Einschlusskriterien und wurden in die Synthese aufgenommen. Die Ergebnisse deuten darauf hin, dass die Prävalenzraten feindselig‐hilfloser Gemütszustände in Abhängigkeit vom psychosozialen Risikostatus im Erwachsenenalter zunehmen. Die Ergebnisse zeigen auch, dass die langfristigen Folgen früher Traumata bei Vorliegen eines feindselig‐hilflosen Gemütszustands schwerer sind, während das Fehlen eines feindselig‐hilflosen Gemütszustands als Schutzfaktor gegen die intergenerationale Weitergabe von Fehlanpassungen wirkt. Schließlich unterstützen die Ergebnisse die diskriminante Validität der Feindselig‐Hilflos‐Klassifikation gegenüber anderen Formen der Bindungsdesorganisation im Erwachsenenalter. Forschungslücken und zukünftige Forschungsrichtungen werden diskutiert.

## 抄録

無力/敵対的な心性 (Hostile‐Helpless States of Mind) :リスク因子、相関、結果に関するスコーピングレビュー

要旨

慢性的な関係性トラウマは、大人になってから、無力/敵対的な心性Hostile‐Helpless States of Mindと呼ばれる、広範に統合されていない愛着形成につながる可能性がある。このような無秩序な愛着型をもつ成人は、養育者に対する評価が相反し、トラウマとなった幼少期の経験を振り返ることが困難であると証明されている。このスコーピングレビューは、無力/敵対的な心性に関するすべての経験的研究の結果を系統的に統合し、この概念の科学的および臨床的な貢献を明らかにし、今後の研究の指針とすることを目的とした初めてのものである。Arksey and O'Malley (2005) の方法論的枠組みに従い、3つのデータベース (PsycArticles、Psychology and Behavioral Sciences Collection、ProQuest) で相互参照キーワードを検索した。その結果、19の研究が対象基準を満たし、統合に含まれることになった。結果は、無力/敵対的な心性の有病率は、成人の心理社会的リスク状態の関数として増加することを示唆している。また、早期トラウマの長期的影響は、無力/敵対的な心性がある場合に大きく、一方、無力/敵対的な心性がない場合は、リスクの世代間伝達に対する防御因子として作用することが明らかになった。最後に、成人の他の愛着障害と無力/敵対的な心性分類の識別妥当性を支持する結果が得られた。研究ギャップと今後の研究の方向性について議論する。

## 摘要

慢性关系创伤可导致成年期普遍的不协调依恋表现的形成, 称为“敌对无助”心理状态。具有这种类型的依恋紊乱的个体, 对看护者的评价是相互矛盾的, 并且很难反思他们的创伤性童年经历。本范围综述第一次系统地整合了所有关于“敌对无助”心理状态的实证研究结果, 以突出这一概念的科学和临床贡献, 并指导未来的研究。按照Arksey和O'Malley (2005) 的“方法论框架”, 在三个数据库 (PsycArticles、Psychology and Behavioral Sciences Collection、ProQuest) 中搜索了交叉引用关键词。总共有19项研究符合纳入标准并被纳入综合研究。结果表明, “敌对无助”心理状态的患病率随着成年人的社会心理风险状况而增加。研究结果还表明, 当存在“敌对无助”心理状态时, 早期创伤的长期后果更大, 而没有“敌对无助”心理状态则可作为一种保护因素, 防止适应不良的代际传递。最后, 研究结果支持“敌对无助”分类对成年期其他形式依恋紊乱的区分效度。本文还讨论了研究的空白和未来的研究方向。

## ملخص

الحالات العقلية المعادية‐العاجزة : استقصاء منهجي لعوامل المخاطرة والارتباطات والعواقب

يمكن أن تؤدي الصدمة العلائقية المزمنة إلى تكوين تمثيلات تعلق غير متكاملة في مرحلة البلوغ ، ويشار إليها على أنها حالات عقلية معادية ‐ عاجزة. الأفراد الذين يعانون من هذا النوع من عدم تنظيم التعلق يُقدمون تقييمات متضاربة لمقدمي الرعاية ويواجهون صعوبة في التعليق على تجارب طفولتهم المؤلمة. هذه المراجعة المنهجية هي الأولى التي تدمج بشكل منهجي نتائج جميع الدراسات التجريبية على الحالات الذهنية المعادية العاجزة في محاولة لتسليط الضوء على المساهمات العلمية والاكلينيكية للمفهوم وتوجيه البحث المستقبلي. ووفقاً لإطار Arksey and O'Malley (2005) المنهجي ، تم البحث عن الكلمات الرئيسية المرجعية في ثلاث قواعد بيانات (PsycArticles ، مجموعة علم النفس والعلوم السلوكية ، ProQuest). في المجموع ، حققت 19 دراسة معايير الاشتمال وتم تضمينها في التحليل. تشير النتائج إلى أن معدلات انتشار الحالات الذهنية المعادية العاجزة تزداد بالارتباط مع حالة الخطر النفسي والاجتماعي للبالغين. تكشف النتائج أيضًا أن العواقب طويلة المدى للصدمة المبكرة تكون أكبر في وجود حالة ذهنية معادية عاجزة ، في حين أن غياب الحالة الذهنية المعادية العاجزة يعمل كعامل وقائي ضد انتقال سوء التكيف بين الأجيال. أخيرًا ، تدعم النتائج الصلاحية التمييزية لتصنيف "معاد‐عاجز" بالمقارنة مع الأشكال الأخرى من عدم تنظيم التعلق في مرحلة البلوغ. تناقش الدراسة الفجوات البحثية واتجاهات البحث المستقبلية.

## 1 INTRODUCTION

Adults’ childhood attachment experiences are evaluated and organized into a state of mind, one that affects how they will perceive and respond to interpersonal relationships, especially the parent‐child relationship (Main et al., 1985; van IJzendoorn, 1995). The results of different meta‐analyses reveal that parents’ attachment state of mind (or attachment representations) influence both parental caregiving behaviors and the quality of parent‐child interactions (Madigan, Bakermans‐Kranenburg, et al., 2006; van IJzendoorn, 1995; Verhage et al., 2018, 2016). Parents with a secure/autonomous state of mind are generally more sensitive and responsive to their child's signals and needs, which promotes child attachment security (Main et al., 1985; van IJzendoorn, 1995; Verhage et al., 2016). However, not all adults are able to provide a balanced and coherent narrative of their attachment experiences, to reflect on these experiences, and to attribute value to attachment relationships (Crowell et al., 2008; Hesse, 2016), resulting in either an insecure or disorganized adult attachment state of mind.

Disorganized attachment states of mind originate from unresolved past traumatic events or interpersonal experiences (Bailey et al., 2007; Main & Hesse, 1990) and increase the risk of having children with a disorganized attachment (Madigan, Bakermans‐Kranenburg, et al., 2006; van IJzendoorn, 1995). Researchers investigating the intergenerational transmission of attachment disorganization have provided a theoretical model based on attachment theory, in which dysregulated parenting behaviors are proposed as the explanatory mechanism (Hesse & Main, 2006; Main & Hesse, 1990). The hypothesis is that parents’ unresolved traumatic experiences impede their ability to respond to their child's emotional cues, resulting in frightened or frightening or extremely insensitive behaviors toward the child (Hesse & Main, 2006; Main & Hesse, 1990). When children perceive their caregivers as frightened or frightening, it places them in an unsolvable dilemma of whether to approach or move away from their caregiver perceived as both a threat and a source of protection (Main & Hesse, 1990). The exposure to frightened or frightening parenting behaviors as well as the failure of the parent to terminate the child's activation of the attachment system contribute to create a chronic hyperarousal of the attachment system that leads to attachment disorganization (Cyr et al., 2010). The results of several studies support this theoretical model, showing significant associations between unresolved trauma/disorganized attachment states of mind, frightened/frightening or dysregulated caregiving behaviors and infant/child attachment disorganization (Jacobvitz et al., 2006, 2011; Lyons‐Ruth et al., 1999; Madigan, Bakermans‐Kranenburg, et al., 2006, Madigan, Moran, et al., 2006; Schuengel et al., 1999).

**KEY FINDINGS**

1. Researchers examining the intergenerational transmission of disorganized attachment from the perspective of Hostile‐Helpless states of mind found that children of parents with Hostile‐Helpless states of mind are at risk of child attachment disorganization.
2. Findings from this scoping review reveal that Hostile‐Helpless states of mind are associated with disruptions in parent‐child interactions and predict maltreating parenting behaviors. They support the need of helping parents recognize how their past experiences and current state of mind may influence the quality of the relationship with their child.
3. The results of several studies show associations between Hostile‐Helpless states of mind and childhood trauma. Interventions should focus on giving adults with a Hostile‐Helpless state of mind the opportunity to reflect on their childhood experiences, recognize the emotions associated with past trauma and address inconsistencies in their internal working models.

**STATEMENT OF RELEVANCE**

Hostile‐Helpless states of mind, a particular form of disorganized attachment in adulthood, can be observed in adults who have experienced chronic relational trauma during childhood. Findings from this scoping review reveal that Hostile‐Helpless states of mind are associated with the quality of parent‐child interactions, atypical caregiving behaviors, and child attachment disorganization. They highlight the need to implement preventive interventions in order to reduce the intergenerational transmission of maladaptation.

To date, studies investigating disorganized attachment representations in adulthood have primarily considered unresolved (U) states of mind in relation to loss or trauma, either alone or in combination with the Cannot Classify (CC) classification^1^. The U classification is assigned to adults who show lapses in reasoning or discourse when discussing specific experiences of abuse and/or loss (Hesse, 2016). Although indicators of disorganization can be identified through the analysis of discourse regarding these specific experiences, this conceptualization of attachment disorganization might be less effective in identifying the more global representational distortions seen in individuals who have experienced chronic relational trauma in childhood (Lyons‐Ruth, Yellin, et al., 2005). The use of the U classification has also failed to fully explain the correspondence between parental state of mind and child attachment disorganization, which van IJzendoorn refers to as the “transmission gap*”* (van IJzendoorn, 1995). As a result, a second conceptualization of disorganized states of mind was proposed as a means of identifying variations in the mental representations of high‐risk and clinical populations, with the potential to further explain the relation between parental attachment and child disorganization (Lyons‐Ruth, Yellin, et al., 2005). Through a comprehensive analysis of the psychological characteristics of clinical populations, Lyons‐Ruth and colleagues developed the Hostile‐Helpless (HH) coding system that is “designed to detect more pervasively unintegrated states of mind that accompany experiences of relational trauma, including the cumulative traumas of consistently hostile or withdrawn parenting, as well as the episodic traumas of abuse events” (Lyons‐Ruth, Yellin, et al., 2005, p.19). Although both classification schemes are applied to the Adult Attachment Interview (AAI; George et al., 1985/1996), and despite the complementary nature of the U and HH classifications, there are important conceptual differences to consider. In contrast to the U coding system, the HH coding system incorporates several psychological processes observed in clinical populations. The defensive functions covered (e.g., splitting) make it a particularly suitable system for identifying the mental representations of individuals who have experienced chronic relational trauma (Lyons‐Ruth, Yellin et al., 2005). Another distinctive feature of the HH coding system is that it considers all parts of the interview, as opposed to specific portions relating to past experiences of loss or abuse. This allows the identification of more global and pervasive representational distortions that appear throughout the individual's entire narrative. Moreover, by considering both episodic and pervasive cumulative traumas, the HH coding system offers the advantage of capturing distorted mental representations that arise from repeated patterns of disruptive parent‐child interactions during childhood. It may therefore be helpful in identifying individuals at risk of experiencing pervasive parenting difficulties (Finger, 2006; Melnick et al., 2008). Finally, as suggested by Bernier and Meins (2008), the HH classification may be more reflective of a characteristic trait of the person as it captures a pervasive lack of integration in the adults’ representations of attachment experiences, whereas the U classification may capture a more temporary state of unintegration that arises strictly when the person discusses past events of abuse and/or loss. Further studies are needed to understand how these two classification systems contribute to our understanding of attachment disorganization in adulthood, particularly in high‐risk populations. The purpose of this paper is to systematically review the current body of knowledge on HH states of mind, both independently and in relation to U or U/CC states of mind.

### 1.1 Hostile‐helpless states of mind

Individuals with a HH state of mind have unintegrated representations of their attachment figures and demonstrate conflicting mental contents during the AAI (Lyons‐Ruth et al., 1995/2006/2011). These adults describe traumatic childhood experiences, but have difficulty reflecting and elaborating on the emotions accompanying these experiences (Lyons‐Ruth, Yellin, et al., 2005). Another distinctive feature of adults with a HH state of mind is the tendency to describe a caregiver in a devaluating or derogatory manner, either as globally malevolent or fearful and abdicating in their parental role, while also identifying with this caregiver by adopting similar attitudes and/or behaviors or stating being very close to this caregiver (Lyons‐Ruth & Jacobvitz, 2016). These individuals fail to address these contradictions and often remain trapped in very intense feelings of fear, rage, or guilt towards their attachment figures. Although transcripts may contain a combination of hostile and helpless indicators, some transcripts are more consistent with the characteristics of a single stance (either hostile or helpless; Lyons‐Ruth & Jacobvitz, 2016).

Adults with a predominantly hostile state of mind represent at least one of their attachment figures in globally devaluating terms, while at the same time presenting some positive evaluations of their caregiver, without acknowledging these contradictions (Lyons‐Ruth et al., 1995/2006/2011). They also tend to portray themselves as being tough, invulnerable or aggressive and describe a similar pattern of behaviors in their attachment figure with whom they identify (Lyons‐Ruth, Yellin, et al., 2005). Signs of minimization and/or dissociation may also be evident when discussing attachment‐related experiences (Lyons‐Ruth et al., 1995/2006/2011). In contrast, adults with a predominantly helpless state of mind tend to identify with an abdicating parental figure, often portrayed as helpless and/or fearful (Lyons‐Ruth & Jacobvitz, 2016). They differ from adults in the hostile subtype in their ability to speak openly about their feelings, while struggling to make sense of past experiences and cope with intense feelings of shame, guilt, or a sense of badness or unworthiness (Lyons‐Ruth, Yellin, et al., 2005). Their narratives are often infused with feelings of fear, and some describe engaging in a protective or caregiving role toward their parent as children (Lyons‐Ruth et al., 1995/2006/2011). A mixed subcategory is assigned to individuals who exhibit characteristics of both a hostile and helpless state of mind. However, most studies use the HH scaled score or the dichotomous HH/non‐HH score in their analyses, without considering subcategories.

A series of studies on HH states of mind have been published in recent years and reveal the discriminating power of this new coding system as a means of identifying those who have experienced severe forms of relational trauma in childhood and who show signs of global representational distortions when discussing past and current attachment relationships. Results from studies investigating child attachment disorganization, parent‐child interactions, and parenting behaviors in association with HH states of mind suggest that this form of attachment disorganization may be involved in the intergenerational transmission of maladaptation. However, these results have yet to be systematically reviewed before general conclusions can be drawn. Mapping the evidence and integrating findings will not only inform future research in the field of attachment disorganization but also provide guidelines for clinical interventions.

### 1.2 Objectives

The purpose of this paper is to (1) synthesize and disseminate research findings on HH states of mind, (2) identify research gaps, and (3) make recommendations for future research. Specifically, this review aims to map the extent of research on HH states of mind by extracting prevalence data as well as information on precursors of HH states of mind. Another aim is to understand how HH states of mind may interfere with adults’ psychological functioning and social relationships, particularly the parent‐child relationship, and how it may have implications for the next generation. Finally, this paper seeks to explore the distinct contributions of HH and U or U/CC states of mind in understanding attachment disorganization in adulthood.

## 2 METHODS

Given the heterogeneous nature of studies on HH states of mind, the scope of research questions that have been examined thus far, and the absence of a comprehensive review of this body of literature, a scoping review is the most appropriate study design. This type of review seeks to provide an overview of the available literature and to identify research gaps in order to suggest future research directions (Peters et al., 2015).

This scoping review was conducted in accordance to Arksey and O'Malley's (2005) Methodological Framework, which has been cited more than 9,000 times. This framework is divided into five stages, namely (1) identifying the research question; (2) identifying relevant studies; (3) study selection; (4) charting the data; and (5) collating, summarizing, and reporting the results (Arksey & O'Malley, 2005). We used Endnote and Covidence for screening and selecting articles. The latter is an online tool that can be used for a variety of reviews and is based on Cochrane Community standards.

### 2.1 Search methods

The search was conducted through several databases to identify empirical studies and doctoral dissertations. An e‐mail was also sent to each author who had published a doctoral dissertation to verify if the results had been published (or were in the process of being published) in a scientific journal. Had the results been published in both a doctoral dissertation and a scientific article, only the data from the article were considered. Finally, reference lists of included articles were screened to ensure that all relevant studies were identified. We used the following keywords and search strategy: (hostile‐helpless OR hostile/helpless) AND (adult* OR mother* OR parent* OR women OR maternal OR men OR father* OR paternal) AND (attachment OR disorgani* OR Adult Attachment Interview OR AAI). All searches were run on December 31, 2020. The search protocol was peer reviewed by two judges (TM/DSL) prior to conducting the search. Keywords were adapted according to the recommendations made by both judges and consultations were held throughout the entire review process to ensure consistency. The revised search strategy was then applied to three databases, from their inception to the end of 2020: (1) PsycArticles, (2) Psychology and Behavioral Sciences Collection, and (3) ProQuest. These databases were selected because they cover empirical research in the field of attachment.

### 2.2 Inclusion and exclusion criteria

The most important criterion for inclusion of studies was the presence and use of the HH coding system developed by Lyons‐Ruth et al. (1995/2006/2011). Because this system is strictly used in conjunction with the AAI, and in order to maintain consistency among the studies selected for the scoping review, we rejected studies that used other coding systems to screen for HH behaviors, such as the Assessment of Representational Risk (ARR) Coding System (Sleed, 2014). Whether researchers studied predictors of HH states of mind, correlates, or consequences, whether they used the HH concept as a dependent, independent, or mediator/moderator variable, and whether they measured HH states of mind categorically (HH vs. non‐HH) or continuously (scaled score from 1 to 9)^2^, all variations were accepted. The findings, however, are interpreted in light of these differences.

Empirical studies with a peer review process and doctoral dissertations were included in three languages (i.e., English, French and Italian), given that research teams in Canada, the United States, the United Kingdom, and Italy have been trained by Lyons‐Ruth and colleagues in using the HH coding system. All study designs were accepted, apart from case studies, as the grids used for quality assessment and data extraction were not applicable to this type of design. This resulted in the exclusion of two studies (Isosävi et al., 2019; Lyons‐Ruth & Spielman, 2004).

We decided to include original studies only, and therefore excluded book chapters in which results from empirical studies were reported. Nevertheless, these book chapters were used to extend the search of studies and were helpful in describing the HH concept in a more comprehensive manner. Full texts published or in press before January 1, 2021 were included in this review. Since the HH concept was first developed in the 1990s, all publications included in the synthesis exceed this date. Only studies with a rigorous methodology were retained, and therefore, opinion texts and conference papers were not considered.

### 2.3 Screening

The initial search yielded 284 results. Three hand‐searched references and one in‐press article (now published) shared by one of the doctoral dissertation authors were added to this count. All references were downloaded into Endnote and imported to Covidence for screening. Duplicate references were removed (*k* = 8) and two judges (JT/CU) independently screened the remaining 280 articles based on titles and abstracts. Disagreements (*k* = 6) were resolved by discussion. In case of uncertainty, studies were included for full‐text screening.

**FIGURE 1:**
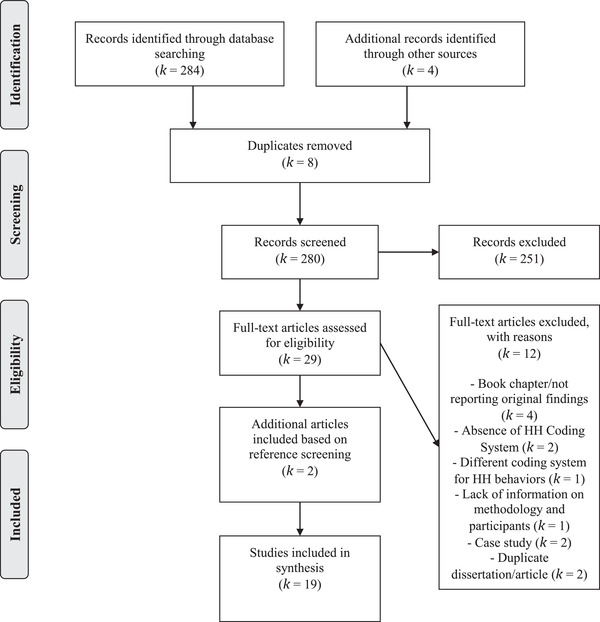
PRISMA flow diagram

Based on inclusion and exclusion criteria, 251 studies were excluded and considered irrelevant to the review, six were included due to uncertainty and the remaining 23 studies were included given their relevance to the review. Interrater agreement was κ = .87. Full‐text assessments were conducted for the remaining 29 references. Three disagreements were resolved after discussion (κ = .77), resulting in the inclusion of 17 relevant references. Reference screening was also conducted to verify if any articles had been missed during the screening process. Two additional references were identified, both of which were published in Italian (Barone & Frigerio, 2009; Guarino et al., 2011). The results of these studies were discussed in one of the authors’ subsequent papers (Frigerio et al., 2013) and summarized in each paper's Abstract. Certain sections of these studies were also translated for data extraction and quality assessment purposes, since the authors of this paper do not speak Italian. In total, 19 references were included in the synthesis (see Figure 1 for PRISMA flow diagram and reasons for exclusion).

### 2.4 Data extraction

In an effort to systematically extract the data, an Excel spreadsheet was created by the team, which was inspired by other spreadsheets used in recent Scoping Reviews. The Excel spreadsheet included 14 categories and was used to extract information related to study aims and design, setting, participants/groups, measures, statistical analyses, as well as relevant findings related to both HH and U or U/CC states of mind. A final category was used to describe the study according to the following classifications: (1) predictor of HH; (2) consequence of HH (for the child or the parent‐child relationship); or (3) correlate of HH (i.e., the direction of the relation was unknown). These three categories were used for the HH states of mind but also in relation to the U or U/CC states of mind when available in the selected studies. Data extraction was completed independently by the same two judges (JT/CU). Interrater agreement was assessed on almost half of the articles (*k* = 9), and the remaining ten were divided between both judges. Interrater agreement was calculated on all 114 completed cells and revealed a high level of agreement (94.74%). In case of uncertainty, the data were reviewed by two independent judges (TM/DSL).

### 2.5 Quality assessment

Quality assessment of studies was conducted using the AXIS appraisal tool (Downes et al., 2016), which consists of 20 questions and is specifically designed to identify potential biases in cross‐sectional studies. As with data extraction, almost half of the articles (*k* = 9) were evaluated by two independent judges (JT/CU), while the ten remaining articles were evenly divided between judges and assessed independently. Disagreements were discussed until consensus was reached. Interrater agreement was calculated on all 180 completed cells and revealed a high level of agreement (95.56%).

## 3 RESULTS

### 3.1 Description of included studies

A summary of the characteristics (aims, setting and design, sample, measures) and main findings of each study (*k* = 19) can be found in Table 1. Although there are 19 studies included in the review, some were published as part of the same research project, resulting in the inclusion of nine independent samples. Most studies included in the synthesis were conducted in the United States (*k* = 10; 53%). The remaining studies were conducted in Italy (26%), Canada (10.5%), and the United Kingdom (10.5%). In addition, most studies were cross‐sectional in design (84%), apart from one longitudinal study (6‐month interval; 5%) and two prospective studies (11%). Most studies were conducted with at‐risk populations. Eight studies included a comparison group (42%), although one of them did not analyze groups separately. Furthermore, a majority of studies (84%) used both the HH State of Mind Coding System and the Adult Attachment Scoring and Classification System (Main et al., 2002) to assess disorganized attachment (HH and U or U/CC states of mind) in adulthood. In terms of sample size, eight studies (42%) had 50 or fewer individuals or parent‐child dyads, three had between 51 and 100 (16%), and eight had more than 100 (42%). HH states of mind were assessed in predominantly female adult samples (>50% of participants), apart from one study in which the participants were predominantly male. Finally, most of the studies were conducted with Caucasian participants (two were conducted with predominantly African American participants). In terms of study classification, seven studies investigated predictors of HH states of mind, such as childhood trauma, 16 investigated the effect of HH states of mind on the child (*k* = 6) or the parent‐child relationship (*k* = 10) and ten investigated correlates of HH states of mind, such as mental health problems or level of sociodemographic risk.

**TABLE 1:** Data extraction of studies included in the synthesis

Authors & year	Setting and design	Aim(s) of study	Sample	Measures related to HH variable	Relevant findings (HH)	Relevant findings (U)	^a^Study classification
Barone et al. (2014)^1^	Italy, cross‐sectional	Examine the effect of descriptive and attachment derived risk factors in predicting filicide.	‐ *N* = 23 Filicidal mothers ‐ Mean age: 34.13 ‐ HH: 65.2%; U/CC: 60.9% ‐ SES: 39.1% low; 56.5% moderate; 4.3% high. ‐ *N* = 37 Mentally ill mothers ‐ Mean age: 33.54 ‐ HH: 27%; U/CC: 27% ‐ SES: 27% low, 64.9% moderate, 8.1% high. ‐ *N* = 61 Normative population ‐ Mean age: 34.11 ‐ HH: 6.6%; U/CC: 14.8% ‐ SES: 8.2% low, 75.4% moderate, 16.4% high	‐ Structural Clinical Interview for DSM‐IV Axis I Disorders. ‐ Traumatic events derived from clinical reports and AAI transcripts. ‐ AAI: traditional coding system and HH coding system.	‐ HH states of mind were found to contribute significantly to the distinction between the mentally ill and filicide groups, after controlling for descriptive and attachment‐based risk factors. ‐ HH states of mind significantly contributed to the prediction of filicide.	‐ U/CC did not contribute significantly to the prediction of filicide.	‐ Consequence for the parent‐child relationship
Barone and Carone (2020)^1^	Italy, cross‐sectional	Examine the effects of HH states of mind and RF in the relation between CA&N and filicide likelihood.	‐ *N* = 23 Filicidal mothers ‐ Mean age: 34.13 ‐ HH: 65.2% ‐ SES: 39.1% low; 56.5% moderate; 4.5% high. ‐ *N* = 23 Non‐Filicidal mothers ‐ Mean age: 35.82 ‐ HH: 21.7% ‐ SES status: 30.4% low; 56.5% medium; and 13.1% high.	‐ Structural Clinical Interview for DSM‐IV Axis I Disorders. ‐ Severity of childhood abuse (7‐point scale derived from CTS‐2, TSS and CTES‐R). ‐ Maternal childhood history of separation or loss (3‐point scale). ‐ AAI: HH coding system and RF coding system.	‐ Higher HH scores mediated the relation between CA&N and filicide likelihood. This relation was further moderated by lower levels of RF.	N/A	‐ Consequence for the parent‐child relationship ‐ Predictor
Barone and Frigerio (2009)^2^	Italy, cross‐sectional	Investigate disorganized attachment as a potential risk factor for parental maltreatment.	‐ *N* = 10 maltreating mothers ‐ Mean age: 27.4 ‐ HH: 70%; U/CC: 40% ‐ SES: 20% no occupation; 60% low; 20% moderate ‐ *N* = 10 non‐maltreating mothers ‐ Mean age: 35.1 ‐ HH: 10%; U/CC: 20% ‐ SES status: 40% low; 60% moderate	‐ AAI: traditional coding system and HH coding system.	‐ Mothers in the maltreating group had higher proportions of HH states of mind compared to mothers in the control group.	‐ Both groups did not differ according to the U/CC versus non‐U/CC classification.	‐ Consequence for the parent‐child relationship
Brumariu et al. (2013)^3^	USA, cross‐sectional	Examine the association between anxiety disorders and quality of attachment and peer relationships.	‐ *N* = 109 young adults and their mothers (30 had no Axis I diagnostic; 44 anxiety disorder; 35 Axis I disorder other than anxiety). ‐ Mean age: 19.9 ‐ Female (young adults): 60.55% ‐ Young adults’ mean HH scaled score: 4.07 to 5.29 ‐ Mean overall insecure/disorganization scaled score (scaled score from 1 (autonomous/secure) to 3 (U/CC): 1.53 to 1.95 ‐ SES: low to moderate	‐ Structural Clinical Interview for DSM‐IV Axis I Disorders. ‐ Goal‐Corrected Partnership in Adolescence Coding System. ‐ AAI: traditional coding system and HH coding system.	‐ Young adults with anxiety disorders had higher levels of HH states of mind compared to young adults with no Axis I diagnosis. This relation was non‐significant for young adults with other Axis I disorders, but no anxiety.	‐ Young adults with anxiety disorders had higher levels of overall insecurity/disorganization compared to young adults with no Axis I diagnosis. This relation was non‐significant for young adults with other Axis I disorders but no anxiety.	‐ Correlate
Byun et al. (2016)^3^	USA, cross‐sectional	Investigate if attachment disorganization mediates the relation between childhood trauma and dissociation in young adulthood.	‐ *N* = 112 young adults ‐ Mean age: 20 ‐ Female: 60% ‐ HH: 50.9%; U: 19.6% ‐ SES: low to moderate	‐ Socioeconomic risk (score from 0–3) ‐ Dissociative Experiences Scale. ‐ Severity of childhood abuse (7‐point scale derived from CTS‐2, TSS and CTES‐R). ‐ AAI: traditional coding system and HH coding system.	‐ Higher levels of HH states of mind were associated with higher levels of socioeconomic risk, lower education level, and higher levels of dissociative symptoms. ‐ HH states of mind were associated with severity of abuse. ‐ HH states of mind did not mediate the relation between childhood trauma and dissociation.	‐ Higher levels of U loss or trauma were not related to dissociative symptoms. ‐ Only U trauma was associated with severity of abuse. ‐ U trauma did not mediate the link between childhood trauma and dissociation.	‐ Correlate ‐ Predictor
Finger (2006)^4^	USA, cross‐sectional	Examine if attachment representations and psychopathological symptoms are related to infant attachment disorganization.	‐ *N* = 62 mothers in methadone treatment ‐ Mean age: 32.36 ‐ HH: 56%; U: 50% ‐ SES: low ‐ *N* = 87 mothers from the community ‐ Mean age: 26.82 ‐ HH: 39%; U: 50% ‐ SES: low	‐ Ainsworth Strange Situation procedure. ‐ The Millon Clinical Multiaxial Inventory‐III. ‐ The Dissociative Experiences Scale. ‐ History of trauma (11 trauma categories assessed by five trauma questionnaires). ‐ AAI: traditional coding system and HH coding system.	‐ HH states of mind were significantly associated with infant disorganization and cumulative child and adult trauma. ‐ Child trauma was a better predictor of HH states of mind than adult trauma. ‐ Methadone using mothers had higher proportions of HH states of mind compared to mothers in the comparison group. ‐ HH states of mind were significantly related to Antisocial, Sadistic, Masochistic, Schizotpal, and Borderline personality disorder scales.	‐ U was not significantly associated with infant disorganization. ‐ Only U Abuse (and not U Loss) was significantly associated with maternal HH states of mind. ‐ Methadone using mothers had higher mean U scores.	‐ Correlate ‐ Predictor ‐ Consequence for the child
Finger et al. (2015)^3^	USA, cross‐sectional	Examine the relation between attachment disorganization in adulthood and BPD and ASPD features.	‐ *N* = 103 young adults ‐ Mean age: 19.9 ‐ Female: 67% ‐ Mean HH scaled score: 4.72 ‐ Mean U scaled score: 2.44 ‐ SES: low to moderate	‐ Structural Clinical Interview for DSM‐IV Axis II Disorders. ‐ Socioeconomic risk (score from 0–3) ‐ Severity of childhood abuse (7‐point scale derived from CTS‐2, TSS and CTES‐R). ‐ AAI: traditional coding system and HH coding system.	‐ HH states of mind were associated with severity of abuse, BPD and ASPD symptoms, the presence of an anxiety disorder, and demographic risk. ‐ HH states of mind mediated the relation between severity of abuse and BPD or ASPD features.	‐ Only U trauma (not U loss) was related to HH states of mind. ‐ Only U trauma was associated with BPD features. U was not associated with ASPD features nor other disorders (e.g., anxiety, depression, substance abuse). ‐ U did not mediate the relation between severity of abuse and BPD/ASPD features.	‐ Correlate ‐ Predictor
Frigerio et al. (2013)^2^	Italy, cross‐sectional	Investigate HH states of mind among a low‐risk sample and two at‐risk samples of women.	‐ *N* = 67 Low‐risk sample ‐ Mean age: 35.5 ‐ Female: 100% ‐ HH: 9%; U/CC: 15% ‐ SES: middle‐high ‐ *N* = 20 Poverty sample ‐ Female: 100% ‐ HH: 20%; U/CC: 10% ‐ SES: low ‐ *N* = 15 Maltreatment risk sample ‐ Female: 100% ‐ HH: 60%; U/CC: 33%	‐ AAI: traditional coding system and HH coding system.	‐ Mothers from the maltreatment risk sample had higher levels of HH states of mind compared to the low‐risk sample and poverty sample. ‐ The following codes distinguished women with a HH state of mind from	‐ HH and U classifications were only moderately associated. ‐ The three samples did not significantly differ in terms of the U/CC classification.	‐ Consequence for the parent‐child relationship
Frigerio et al. (2013)^2^					women without a HH state of mind: *Global Devaluation of a Hostile Caregiver, Identification with a Hostile Caregiver; Identification with a Helpless Caregiver, Ruptured Attachments in Adulthood; Affect Driven Confused Speech, Blocking out*.		
Guarino et al. (2011)^5^	Italy, cross‐sectional	Evaluate the effect of maternal HH states of mind on the quality of mother‐child interactions.	‐ *N* = 20 mother‐child dyads at risk for maltreatment ‐ Mean age (mothers): 27.6 years ‐ Mean age (children): 24.7 months ‐ Female (children): 45% ‐ HH (mothers): 40%; U/CC (mothers): 35% ‐ SES: 60% of women unemployed, 25% of the sample reported seven to eight risk factors, 50% had between five and six, and one person (10%) had three risk factors	‐ Feeding Scale‐Observational Scale for mother‐infant interaction during feeding. ‐ AAI: traditional coding system and HH coding system.	‐ HH mothers demonstrated more difficulty during interactions with their child and more negative affects. ‐ Most women with a HH state of mind had several concomitant risk factors (between 6 and 7). ‐ HH mothers vs. non‐HH mothers differed in terms of *Interactive conflict* and *Affective state of the dyad*, whereas no significant differences emerged in the *Maternal affective state* and *Behaviors of child food refusal* scales.	‐ Of the eight women classified as HH, three were rated U/CC with respect to attachment.	‐ Consequence for the parent‐child relationship ‐ Correlate
Honde (2007)^4^	USA, cross‐sectional	Investigate if disrupted maternal affective communication is related to maternal HH states of mind and infant disorganization.	‐ *N* = 149 mother/infant dyads. ‐ Mothers mean age: 29.12 ‐ HH: 45% ‐ U: 44%	‐ Disrupted maternal affective communication with infant: coded using the AMBIANCE coding system. ‐ Cumulative risk (five risk factors). ‐ AAI: traditional coding system and HH coding system.	‐ Mothers with a HH state of mind had higher levels of disrupted maternal affective communication compared to non‐HH mothers, evidenced by more affective communication errors, role‐boundary confusion, disorientation, and intrusiveness/negativity. ‐ Mothers in the Hostile subtype showed more intrusiveness/negativity. Mothers in the Mixed or Helpless subtypes showed more role boundary confusion and disoriented behavior. ‐ HH states of mind were associated with infant disorganization even after controlling for contextual risk factors.	‐ U states of mind were not related to higher levels of disrupted affective communication.	‐ Consequence for the child ‐ Consequence for the parent‐child relationship
Lyons‐Ruth et al. (2007)^6^	UK, cross‐sectional	Examine if women with BPD differ from women with dysthymia in terms of HH states of mind.	‐ *N* = 12 borderline patients ‐ Mean age: 35.2 ‐ Female: 100% ‐ HH: 100%; U: 75% ‐ SES: social class 1 to 5 ‐ *N* = 11 dysthymic patients ‐ Mean age: 32.4 ‐ Female: 100% ‐ HH: 55%; U: 17% ‐ SES: social class 1 to 5	‐ Occurrence of physical or sexual abuse to age 16 (coded from the AAI). ‐ AAI: traditional coding system and HH coding system.	‐ Three HH indicator codes (i.e., *globally devaluing representations, Identification with a hostile caregiver, Punitive or caregiving stance towards parents in childhood*) were more frequent among BPD patients compared to dysthymic patients.	‐ A moderate association was found between the HH and U classifications. ‐ Only one HH indicator code (i.e., *sense of self as bad*) was associated with the U classification.	‐ Correlate
Lyons‐Ruth et al. (2003)^3^	USA, longitudinal	Examine the relations between childhood trauma, maternal states of mind and infant disorganization.	‐ *N* = 45 mothers and their children ‐ Female (children): 38% ‐ HH (mothers): 51% ‐ SES: low	‐ Demographic risk (sum of six factors). ‐ Ainsworth Strange Situation procedure (at 12 and 18 months). ‐ Severity of childhood trauma (5‐point scale). ‐ Maternal history of separation or loss (4‐point scale). ‐ AAI: traditional coding system and HH coding system.	‐ HH levels were not significantly associated with demographic risk factors but were related to the severity of mothers’ childhood trauma. ‐ Mothers exposed to violence and sexually abused mothers differed significantly from those not exposed to violence in overall HH status. ‐ HH state of mind was the only significant predictor of infant disorganization at 18 months, which was not the case at 12 months.	‐ The HH and U classifications were not related. ‐ Only severity of parental U loss (not U trauma) was associated with HH states of mind. ‐ Maternal U state of mind significantly contributed to infant disorganization at 12 months, but not at 18 months.	‐ Consequence for the child ‐ Predictor ‐ Correlate
Lyons‐Ruth, Yellin, et al. (2005)^3^	USA, cross‐sectional	Develop and validate the HH coding system in the hopes of identifying additional predictors of infant disorganization.	‐ *N* = 45 mothers and their children ‐ Female (mothers): 100% ‐ Female (children): 38% ‐ HH (mothers): 51%; U (mothers): 29%; CC (mothers): 13% ‐ SES: low	‐ Demographic risk (sum of five factors). ‐ Ainsworth Strange Situation procedure. ‐ Disrupted maternal affective communication with infant: coded using the AMBIANCE coding system. ‐ AAI: traditional coding system and HH coding system.	‐ HH levels were not significantly associated with demographic risk factors. ‐ HH states of mind were significantly associated with infant disorganization and maternal disrupted affective communication with the infant. The relation between HH states of mind and infant disorganization was non‐significant after controlling for disrupted communication.	‐ The HH and U or U/CC classifications were not related. ‐ Maternal U states of mind were not significantly associated with the level of infant disorganized attachment behavior but were marginally associated with disrupted affective communication.	‐ Consequence for the child ‐ Consequence for the parent‐child relationship ‐ Correlate
Milot et al. (2014)^7^	Canada, cross‐sectional	Examine HH states of mind among neglecting and at‐risk of neglecting mothers.	‐ *N* = 70 neglecting mothers and at‐risk of neglecting mothers. ‐ Mean age: 29 ‐ HH: 64% (mean scaled score: 6.7) ‐ SES: low	‐ The Childhood Trauma Questionnaire ‐ short version. ‐ AAI: HH coding system.	‐ Neglecting mothers and at‐risk of neglect mothers did not differ in terms of HH states of mind. ‐ Childhood trauma was more prevalent among HH mothers than non‐HH mothers. Mothers who experienced more forms of maltreatment had higher HH scores.	‐ N/A	‐ Predictor
Milot et al. (2014)^7^					‐ HH mothers reported significantly more childhood abuse (emotional, sexual, physical) and neglect (physical) than non‐HH mothers.		
Obsuth et al. (2014)^3^	USA, cross‐sectional	Examine the link between disorganized states of mind and young adults’ behaviors during interactions with their mothers.	‐ *N* = 120 young adults and their mothers ‐ Mean age: 19.9 ‐ Female (young adults): 57.5% ‐ HH (young adults): not specified; U (young adults): 16.7% (*n* = 114) ‐ SES: predominantly low	‐ Goal‐Corrected Partnership in Adolescence Coding System. ‐ AAI: traditional coding system and HH coding system.	‐ Young adults’ HH states of mind were significantly related to collaboration and young adults’ punitive behavior toward the parent, which was not the case for Disorientation or Caregiving/Role Confusion. ‐ After controlling for punitive interaction, collaboration was not a significant predictor of HH states of mind. ‐ After controlling for collaboration, punitive interaction was not a significant predictor of HH states of mind.	‐ Young adults’ U states of mind were significantly related to collaboration. ‐ U states of mind were not significantly associated with punitive control or Caregiving/Role Confusion.	‐ Correlate
Sauvé et al. (2021)^8^	Canada, cross‐sectional	Investigate whether HH states of mind moderate the association between parents’ past trauma and child behavior problems.	‐ *N* = 61 parent‐child dyads whose child was maltreated or at high‐risk of maltreatment ‐ Child mean age: 41.7 months ‐ Parental mean age: 27 years ‐ Female (children): 36% ‐ Female (parents): 95% ‐ HH (parents): 66% ‐ SES: 68% below 25,000$/year, 24% between 25,000 and 50,000$/year, 8% at or above 50,000$/year.	‐ Child Behavior Checklist questionnaire. ‐ Childhood Trauma Questionnaire‐short version. ‐ AAI: HH coding system.	‐ Severe childhood trauma was more prevalent among parents with a HH state of mind compared to parents without a HH state of mind. ‐ HH states of mind significantly predicted externalizing child behaviors, which was not the case for internalizing problems. ‐ Parental HH states of mind moderated the relation between parents’ history of trauma and child behavior problems (both externalizing and internalizing). ‐ The severity of parents’ childhood trauma was associated with the extent of child behavior problems, but only for parents in the HH group.	N/A	‐ Consequence for the child ‐ Predictor
Terry et al. (2021)^9^	UK, cross‐sectional	Examine the link between maternal HH states of mind assessed during pregnancy and child removal status before age 2 due to maltreatment or risk of maltreatment.	‐ *N* = 13 mothers whose infants were removed by social care before the age of 2 ‐ Mean age: 19.8 ‐ Infant age at time of removal: 7.17 months ‐ Mean HH scaled score: 5.7 ‐ SES: low ‐ *N* = 13 mothers whose infants remained in their care throughout the course of the intervention. ‐ Mean HH scaled score: 3.5 ‐ SES: low	‐ Pregnancy Interview—Revised. ‐ AAI: HH coding system (adapted for the Pregnancy Interview).	‐ Higher HH scores were found among mothers whose infant was removed from their custody between the ages of 0 and 2. ‐ Mothers’ HH classification was significantly associated with child removal status.	N/A	‐ Consequence for the parent‐child relationship
Vulliez‐Coady et al. (2013)^3^	USA, prospective	Examine the association between parental disorganized states of mind and role confusion.	‐ *N* = 51 mothers and their young adult child. ‐ Parental mean age: 45. ‐ Young adults’ mean age: 19.9. ‐ Female (young adults): 37.25%. ‐ HH (mothers): 61.8%; U (mothers): 35.3% ‐ SES: low‐income	‐ The Parental Assessment of Role Confusion Scale. ‐ AAI: traditional coding system and HH coding system.	‐ HH states of mind were significantly associated with PARC role confusion: HH mothers had a mean PARC score of 3.99 whereas non‐HH mothers had a mean score of 2.18. ‐ PARC role confusion was significantly associated with Caregiving/Role confusion during mother‐child interactions.	‐ PARC role confusion was not associated with maternal U state of mind. ‐ Higher levels of U loss (not U abuse) were significantly related to more PARC role confusion.	‐ Consequence for the parent‐child relationship
Yellin (2001)^3^	USA, prospective	Examine the relation between parental disorganized states of mind and infant disorganized attachment behaviors.	‐ *N* = 35 mothers and infants. ‐ Mothers mean age at the time of birth of their first child: 21.4. ‐ SES: low‐income. ‐ Female (infants): 45.7%. ‐ Mean HH scaled score (mothers): 5.4. ‐ Mean U scaled score (mothers): 2.9.	‐ Ainsworth Strange Situation procedure (at 18 months). ‐ Frightened/Frightening maternal behavior. ‐ Disrupted maternal affective communication with infant: coded using the AMBIANCE coding system. ‐ Demographic risk (sum of six factors). ‐ Center for Epidemiological Studies Depression scale. ‐ AAI: traditional coding system and HH coding system.	‐ HH levels were not significantly associated with infant disorganization. ‐ HH levels did not differ between the disorganized and organized groups of children. ‐ There was no significant relation between HH levels and maternal behavior on either of the coding systems. ‐ The four groups of mothers (U, not HH; HH, not U; U and HH; neither U nor HH) did not differ significantly in levels of risk, depression, or infant disorganization.	‐ U state of mind was not significantly related to infant disorganization. ‐ Mothers classified differently in terms of U states of mind did not differ significantly in levels of risk, depression, or infant disorganization.	‐ Consequence for the parent‐child relationship ‐ Consequence for the child ‐ Correlate

*Note*. AAI, adult attachment interview; ASPD, antisocial personality disorder; BPD, borderline personality disorder; CA&N, child abuse and neglect; CC, cannot classify; CTES‐R, childhood traumatic experiences scales‐revised; CTS‐2, conflict tactics scale 2nd version; HH, hostile‐helpless; RF, reflective functioning; SES, socioeconomic status; TSS, traumatic stress schedule; U, unresolved.

^a^ For each study, the following classifications were used according to study aims (1) predictor of HH, (2) consequence of HH (for the child or the parent‐child relationship) or (3) correlate of HH.

^1–9^Studies with matching numbers have been conducted with the same sample (either in part or in whole).

In terms of the methodological quality of the reviewed studies (*k* = 19), all were considered reliable as minimal bias was detected. Of the 20 questions included in the AXIS tool, each study scored 16 or higher, representing at least 16 correct answers (e.g., the reference population was clearly defined, the measures used were validated). Information regarding the quality assessment of studies is presented in Supplemental Materials, Table S1.

### 3.2 Hostile‐helpless states of mind and level of psychosocial risk

Based on the analysis of descriptive data, HH prevalence rates appear to increase as the level of risk in populations increases. In normative samples, the proportion of HH states of mind is below 10% (Barone et al. 2014 [*n* = 61]; Frigerio et al. 2013 [*n* = 67]), while it rises to 27% in adults diagnosed with an anxiety or mood disorder (Barone et al. 2014 [*n* = 37]), and to 51% and 100% in adults with personality disorder features (Finger et al. 2015 [*n* = 52]) or a personality disorder diagnosis (Lyons‐Ruth et al. 2007 [*n* = 12]). Among maltreating mothers or mothers at risk of child maltreatment [*n* from 10 to 70], prevalence rates range from 40% to 75% (Barone & Frigerio, 2009; Barone & Carone, 2020; Frigerio et al. 2013; Guarino et al., 2011; Milot et al. 2014; Terry et al., 2021).

### 3.3 Hostile‐helpless states of mind and sociodemographic risk

As part of a longitudinal project involving mother‐child dyads (*N* = 76), a total of four studies documented the relation between HH states of mind and sociodemographic risk variables. Families were followed from infancy (18 months) to late adolescence/young adulthood^3^. Yellin (2001) initially found that there was no link between the level of sociodemographic risk (*N* = 35) and mothers' HH state of mind, which was also observed when the sample included 10 additional participants (Lyons‐Ruth et al., 2003; *N* = 45). In both studies, sociodemographic risk was composed of six factors: (1) African‐American or Hispanic mother; (2) no high‐school diploma; (3) single parent; (4) parenthood before age 20; (5) government assistance; and (6) multiple children under the age of six. Almost 20 years later, children from the original sample (*N* = 76) were contacted and asked to participate in the follow‐up phase of the longitudinal study. Approximately 50 young adults (mean age: 19.9 years) agreed to participate and were matched to other individuals to expand the sample size to over 100 participants (Byun et al., 2016; Finger et al., 2015). These last two studies, using this sample of over 100 young adults, revealed a significant association between young adults’ HH state of mind and the level of sociodemographic risk of the family of origin, assessed according to 3 factors (annual household income below $40 000, mother had no live‐in partner, mother had no post‐secondary education). Considering that all the studies on sociodemographic risk were based on the same original sample, it is likely that the first two studies (Lyons‐Ruth et al., 2003; Yellin, 2001) lacked the statistical power to reveal a significant association.

### 3.4 Hostile‐helpless states of mind and adult psychological functioning

Given that HH states of mind involve a disruption in the subject's mental representations, several researchers examined this variable in relation to adult psychological functioning (*k* = 7). A number of studies found an association between HH states of mind and anxiety/mood disorders. For instance, Barone et al. (2014) revealed that HH states of mind were more prevalent among mentally ill mothers (27%; *n* = 37) than mothers from the normative population (6.6%; *n* = 61). Higher HH indices were also found in young adults who suffer from an anxiety disorder compared to those without any Axis I diagnosis (Brumariu et al., 2013). Yellin (2001), on the other hand, found no relation between low‐income mothers’ HH states of mind and their levels of depression (*N* = 35). Other studies found that HH scores and indicators were related to Borderline Personality Disorder (BPD; Finger et al., 2015; Lyons‐Ruth et al., 2007) and Antisocial Personality Disorder features (ASPD; Finger et al., 2015), as well as to sadistic, masochistic and schizotypal personality disorder traits (Finger, 2006). Interestingly, no significant association was found with ASPD when U indicators were considered instead of the HH scaled scores, and only Unresolved trauma (not Unresolved loss) was related to BPD features (Finger et al., 2015). Lastly, significant associations were found between HH states of mind and dissociative symptoms (Byun et al., 2016) as well as substance abuse (Finger, 2006). Considering the cross‐sectional design of these studies, it is not clear whether HH is a precursor or a consequence of these symptoms. Nevertheless, these findings suggest that having a HH state of mind is significantly related to psychological disorders.

### 3.5 Hostile‐Helpless states of mind and the intergenerational transmission of trauma

The results of several studies show associations between HH states of mind and childhood trauma (*k* = 7), dysfunctional parent‐child interactions (*k* = 5), maltreating parenting behaviors towards the child (*k* = 5), infant/child disorganization (*k* = 4) and child behavior problems (*k* = 1), supporting the hypothesis that HH states of mind may play a role in the intergenerational transmission of maladaptation. These studies represent 84% (16/19) of the references included in the review.

#### 3.5.1 Hostile‐helpless states of mind and childhood trauma

Whether researchers derived a score of childhood trauma from clinical reports, AAI transcripts, multiple self‐reported questionnaires or a single self‐reported questionnaire, and whether they used a dichotomous trauma score (absence vs. presence) or a severity score on a continuous scale, childhood traumatic experiences were significantly associated with HH states of mind in all studies (*k* = 7) investigating this relation. Specifically, HH states of mind have been associated with overall severity of violence, abuse and neglect (Barone & Carone, 2020; Byun et al., 2016, Finger et al., 2015; Lyons‐Ruth et al., 2003; Sauvé et al., 2021), as well as with maternal abuse alone (Finger et al., 2015). Milot et al. (2014) examined the differential effects of five types of maltreatment in a sample of low‐income mothers (*N* = 70) reported for physical neglect (or risk of neglect). Those with higher HH scores more frequently reported experiences of physical neglect and abuse (physical, emotional, and sexual) in their childhood than mothers with lower HH scores. Additionally, more various types of maltreatment were reported in the HH group compared to non‐HH mothers. The results of another study found that childhood trauma is a better predictor of HH states of mind than adult trauma, and that childhood family violence is a better predictor of HH classification than childhood physical abuse (Finger, 2006). Finally, in a study by Lyons‐Ruth et al. (2003), childhood trauma was significantly associated with four HH indicators, that is, *Identification with a Hostile Caregiver*, *Laughter at Pain*, *Global Devaluation of a Caregiver* and *Sense of Self as Bad*.

#### 3.5.2 Hostile‐helpless states of mind and parent‐child interactions

Several studies have investigated whether HH states of mind interfere with parent‐child interactions (*k* = 6). Although most of these studies (*k* = 4) analyzed data from the same longitudinal sample at different points in time, the measures used to assess the quality of interactions vary from one study to another and show the extent to which HH states of mind can be related to various dimensions of the parent‐child relationship. In a study by Lyons‐Ruth, Yellin, et al. (2005), mothers’ HH states of mind were significantly related to disrupted parent‐infant communications (*N* = 45), a finding that did not reach significance when the sample size was smaller (*N* = 35; Yellin, 2001). Interactions were coded using the Atypical Maternal Behavior Instrument for Assessment and Classification observation grid (AMBIANCE; Bronfman et al., 1999), which is used to screen for disturbances in parent‐child emotional communication. The scale includes five dimensions: (1) errors in affective communication; (2) role confusion; (3) intrusiveness; (4) frightened/disoriented behaviors; and (5) withdrawal behaviors. In Lyons‐Ruth, Yellin, et al.’s study (2005) study, only a marginal association was found between disrupted affective parent‐infant communications and mothers’ U states of mind. This relation decreased when both U and CC were considered. Participants in these studies were followed longitudinally to replicate the findings once they had reached young adulthood (mean age: 19.9). In one study, mothers in the HH group had scores almost twice as high as mothers in the non‐HH group on a representational measure of parental role confusion which, in turn, was associated with caregiving/role‐confusion behaviors during dyadic young adult‐mother interactions (Vulliez‐Coady et al., 2013). Conversely, the association between role confusion and mother's U state of mind was non‐significant (Vulliez‐Coady et al., 2013). In another study, Obsuth et al. (2014) examined the association between young adults' HH states of mind and how they engage in interactions with their mother in a non‐structured task as well as when discussing a topic of disagreement. Interactions were coded using the Goal‐Corrected Partnership in Adolescence Coding System (GPACS; Lyons‐Ruth, Hennighausen, et al., 2005), which measures the young adult's punitive and caregiving control towards the parent. Results showed that young adults in the HH group displayed significantly less collaboration and more punitive control when interacting with their mother compared to non‐HH participants.

In another study, Honde (2007) examined the quality of interactions in a high‐risk sample of 149 mother‐infant dyads who were predominantly African American. Findings also revealed that mothers in the HH group had higher scores for disrupted affective communications on the AMBIANCE measure, evidenced by more affective communication errors, role‐boundary confusion, disorientation, and intrusiveness/negativity when interacting with their child. These findings did not reach significance in relation to U states of mind. Additional analyses revealed that the hostile subtype was associated with greater intrusiveness/negativity, whereas the mixed/helpless subtype was associated with greater role/boundary confusion and dissociative/disorganized behavior during mother‐infant interactions (Honde, 2007). Finally, a study conducted with 20 Italian mother‐child dyads revealed that mothers with a HH state of mind exhibited more difficulties and negative affects during interactions with their child (mean child age: 24 months; Guarino et al., 2011).

#### 3.5.3 Hostile‐helpless states of mind and maltreating parenting behaviors

Given that HH states of mind have been linked to both traumatic childhood experiences and disrupted parent‐child interactions, researchers examined whether they might also be implicated in child maltreating behaviors. Frigerio et al. (2013) investigated the prevalence of HH states of mind in a low‐risk sample and two at‐risk samples and found that women in the maltreatment risk group had higher levels of HH states of mind than women from both the poverty sample and the low‐risk sample. Women in the maltreatment risk group were also more likely to be classified as HH than women in the other two groups, a finding that was found to be non‐significant using the U/CC classification. Higher proportions of HH states of mind were also found in a sample of mothers (*n* = 10) monitored by child protection services compared to mothers (*n* = 10) in the control group (Barone & Frigerio, 2009). Another study reported high prevalence rates in neglecting mothers and mothers at‐risk of neglect (Milot et al., 2014 [*N* = 70]). More recently, Terry et al. (2021) examined the relation between HH states of mind and the removal from home of children monitored by Child Protection Services. Mothers’ HH state of mind was coded from the Pregnancy Interview (Slade, 2011), using an adapted version of the HH coding system. The authors found that mothers' HH status, assessed during pregnancy, was significantly associated with child removal prior to age 2. These results were significant whether the HH variable was measured continuously or categorically. In another cross‐sectional study conducted in Italy, Barone et al. (2014) examined the contribution of different risk factors in the study of filicide, the homicide of a child by a parent. The sample of 121 women included three groups: women from the general population, women with a mental health diagnosis, and women who had committed a filicide. Results showed a higher proportion of HH states of mind in the filicidal and mental illness groups. In the filicidal group, HH states of mind were identified in 65.2% of mothers. Findings reveal that the combination of HH states of mind and risk factors (e.g., low socioeconomic status, past traumatic events, mental health diagnosis) significantly contributed to predicting filicide, whereas the combination of mothers’ U/CC states of mind and risk factors did not. More recently, Barone and Carone (2020) used a subsample of this first study to examine the mediating effects of HH states of mind and reflective functioning in the relation between childhood maltreatment and the risk of filicide. They found that mothers' HH states of mind mediated this relation, which was further amplified by lower levels of reflective functioning.

#### 3.5.4 Hostile‐helpless states of mind and child attachment and adaptation

Five studies have examined the intergenerational transmission of disorganized attachment from the perspective of HH states of mind, three of which were conducted with the same sample (Lyons‐Ruth et al., 2003; Lyons‐Ruth, Yellin, et al., 2005; Yellin, 2001). Lyons‐Ruth et al. (2003 [*N* = 45], 2005 [*N* = 45]) found that only mothers’ HH states of mind, as opposed to U states of mind, were a significant predictor of child disorganization at 18 months. The study revealed that this relation was partly mediated by a disturbed affective communication between the mother and her child (Lyons‐Ruth, Yellin, et al., 2005). When the sample size was smaller (*N* = 35) no significant results were found (Yellin, 2001).

The relation between maternal HH states of mind and child/infant attachment disorganization was also found to be significant in at‐risk and ethnic‐minority samples (Finger, 2006; Honde 2007). Conversely, the relation between mothers’ U states of mind and disorganized infant attachment was non‐significant (Finger, 2006). This study also investigated the association between maternal HH subtypes (hostile vs. mixed/helpless) and disorganized infant attachment subtypes, but found no significant relation (Finger, 2006). In a recent Canadian study, Sauvé et al. (2021) examined the relation between parental childhood trauma, HH states of mind, and child behavior problems. In total, 61 parents (95% mothers) of predominantly low socioeconomic status and their child (mean age: 41 months) participated in the study, most of whom (*n* = 50) were recruited through Child Protection Services. Results revealed that the HH classification was a significant predictor of externalizing problems, but not internalizing problems. Furthermore, the HH classification was found to be a significant moderator of the relation between parents’ past trauma and child internalizing and externalizing problems: among parents with a HH state of mind, more severe childhood maltreatment exposure was related to higher levels of child behavior problems, which was not the case among non‐HH parents (Sauvé et al., 2021).

## 4 DISCUSSION

This review aimed to provide a summary of evidence related to HH states of mind and to identify future areas of research. A total of 19 studies conducted in four different countries were included in the review, all of which provide empirical evidence to support the validity of the HH classification system in capturing disorganized attachment representations among various at‐risk populations. Individuals with a HH state of mind have difficulty integrating their negative childhood attachment experiences in a coherent manner, resulting in contradictory evaluations of attachment figures during the AAI (Lyons‐Ruth & Jacobvitz, 2016). For example, a person may report being very close to an attachment figure that is described as globally malevolent/hostile or helpless/abdicating. These individuals are unable to reflect on these pervasive contradictions and maintain representations of themselves and others that are tainted by their negative childhood experiences (Lyons‐Ruth et al., 1995/2006/2011).

Results from this scoping review reveal that the HH coding system is particularly effective in detecting disorganized attachment representations among people who have experienced episodes of maltreatment. The HH coding system seems especially adapted to capture the mental representations of individuals from high‐risk and clinical samples who are most at risk of perpetuating the intergenerational cycle of trauma, beyond what is being captured by the U and/or CC classifications (Lyons‐Ruth, Yellin, et al., 2005). With the integration of defensive features (e.g., splitting, laughter at pain) observed in clinical populations, the consideration of both episodic and pervasive cumulative traumas, and the evaluation of the individual's entire narrative rather than specific events of abuse and/or loss, the HH coding system offers the potential to capture features of adult disorganization that have not previously been addressed by other AAI disorganized classifications.

Taken together, findings suggest that severe experiences of childhood abuse and neglect are related to HH states of mind and that the consequences of early relational trauma are most important when combined with HH attachment representations. Indeed, results indicate that HH states of mind most strongly predict parental violence when combined with other risk factors such as low socioeconomic status, mental illness and prior trauma (Barone et al., 2014). A recent study also found that only among parents with a HH state of mind were parents’ severe childhood maltreatment experiences related to internalizing and externalizing problems in children (Sauvé et al., 2021). Other researchers found a mediating effect of HH states of mind on the relation between childhood abuse and psychopathology traits in adulthood (Finger et al., 2015) as well as between trauma exposure and the likelihood of committing filicide (Barone & Carone, 2020), suggesting that interpersonal difficulties cannot be attributed solely to childhood maltreatment experiences, and maybe best understood through the way these childhood experiences affect a person's attachment representations. Several other studies have found that a HH state of mind is predictive of infant attachment disorganization (Finger, 2006; Honde, 2007; Lyons‐Ruth et al., 2003), infant removal from the family by Child Protection Services (Terry et al., 2021), disrupted mother‐infant affective communication (Lyons‐Ruth, Yellin, et al., 2005), as well as maternal role confusion in mothers’ representations of their relationship with their adult child (Vulliez‐Coady et al., 2013). HH states of mind have also been associated with mental health problems, including personality disorder features (Finger et al., 2015), anxiety disorders (Brumariu et al., 2013), and dissociative symptoms (Byun et al., 2016). Results from this scoping review, therefore, suggest that HH states of mind do not simply reflect a symptom or outcome of early traumatic experiences, but also constitute a mechanism by which early trauma leads to important consequences for the adults themselves, their children, and the parent‐child relationship. Conversely, researchers found that the absence of a HH state of mind in parents with a history of trauma constitutes a protective factor for children's social development and can lower the risk of intergenerational continuity of risk (Sauvé et al., 2021), which stresses the importance of early interventions.

### 4.1 Research gaps and future directions

Although there has been an expansion of knowledge associated with HH states of mind in recent years, more longitudinal studies are needed to fully understand the factors, alongside early trauma, that may lead to its development, such as dysfunctional parent‐child interactions, child attachment disorganization and exposure to parental psychological distress. In addition to replicating current findings with larger samples, studies using an ecological approach are particularly critical to further our understanding of the precursors, consequences and correlates of HH states of mind. Identifying risk factors at different levels of the family ecology could result in new attachment‐based interventions aimed at promoting the positive developmental trajectory of children exposed to problematic parenting behaviors. Given the relation between parental HH states of mind and child attachment disorganization, it is important to examine how certain factors such as the quality of the parent‐child relationship may influence the transmission process. Understanding through which mechanisms attachment disorganization is transmitted from one generation to the next may increase the potential of interventions to act on these factors in the hopes of breaking the intergenerational cycle of risk and maladaptation. Interventions should include components that address HH features, and future studies should investigate whether HH states of mind influence the effectiveness of interventions, as has been found for U states of mind (Moran et al., 2008).

Considering the link between parental HH states of mind and difficulties in parent‐child interactions, another avenue of research is to investigate how parents with HH states of mind understand and represent their child's needs. In this regard, it would be relevant to adapt the HH coding system for interviews that focus on parents’ representations of their children, such as the *Parent Development Interview* (Slade et al., 2004). This would provide insight as to how these representations relate to the parent's ability to care for the child. Future studies should also expand their focus to include school‐aged children and adolescents, as all studies on HH states of mind so far have been conducted with parents and young children (≤7 years old) or adults (≥19 years old). Given that children's attachment representations can be assessed in middle childhood, these studies could investigate how attachment is transmitted on a representational level. Similarly, considering the small number of studies on HH states of mind with male participants (*k* = 5/19; 26.3%), more research involving men and fathers is needed, as evidence suggests they influence children's development differently from mothers (Cabrera et al., 2014).

Given that most studies investigating HH states of mind used a cross‐sectional design (*k* = 16), future studies should favor a prospective approach. Studies should also pursue the investigation of the differential effects of HH classification subtypes, that is, whether hostile states of mind lead to different outcomes compared to helpless states of mind. This line of research should be pursued given that most studies investigating HH states of mind in relation to childhood maltreatment experiences, filicide, and child behavior problems in at‐risk populations have reported higher proportions of parents with a Helpless stance compared to a Hostile stance (Barone & Carone, 2020; Milot et al., 2014; Sauvé et al., 2021). Finally, it would be interesting to explore the stability of HH indicators across generations, as well as whether the transmission process occurs according to the same subtype (e.g., hostile to hostile) or a different subtype (e.g., hostile to helpless).

### 4.2 Implications for theory and practice

The results of this review have important implications for theory and research, as they provide additional evidence of how chronic relational trauma during childhood contributes etiologically to the development of adult HH states of mind, and how parental HH states of mind further predict child attachment disorganization. Moreover, findings reveal that attachment disorganization in adulthood not only occurs in the form of Unresolved Loss and/or Trauma but may also take the form of unintegrated attachment representations characterized by incompatible and contradictory evaluations of attachment‐related experiences (Lyons‐Ruth, Yellin, et al., 2005).

The results of this review also provide insight into the contribution of adult attachment representations to adaptive functioning. Specifically, they highlight attachment disorganization, in the form of HH states of mind, as a determinant factor of psychosocial maladjustment and dysfunctional parent‐child interactions. Parents who have experienced chronic relational trauma and have not yet reflected on these emotional experiences and their potential consequences may be more likely to engage in hostile and/or helpless behaviors toward their child, as the child's needs and distress signals may reactivate their own traumatic memories (Hesse & Main, 2006; Terry et al., 2021). The establishment of a HH dyadic parent‐child relational model may, in turn, interfere with the development of the child's self‐regulation abilities and lead to disruptions in child adaptive functioning (Sauvé et al., 2021) and the continuity of attachment disorganization across generations (Lyons‐Ruth et al., 1999).

Regarding implications for practice, results from this review provide additional evidence of the significant impacts of childhood relational trauma and reinforce the importance of early detection and intervention. Specifically, they support the need of (1) identifying individuals with a history of relational trauma, (2) screening for HH mental representations in young adulthood, and (3) implementing preventive interventions in order to foster positive developmental trajectories among vulnerable youth and reduce the intergenerational transmission of maladaptation. Interventions should especially target adolescents and adults prior to their transition to parenthood, as well as expecting parents and young parents. As explained by Lyons‐Ruth et al. (2004), the transition into parenthood can be challenging and overwhelming for adults who have experienced relational traumas in their past, as they must set aside their own needs to meet those of their child and are exposed to their child's daily distress signals which may cause them to relive past traumas. For this reason, interventions should focus on giving adults the opportunity to reflect on their childhood experiences, recognize the emotions associated with past trauma and address inconsistencies in their internal working models (Lyons‐Ruth et al., 2007). Once they become parents, emphasis should be placed on helping parents recognize how their past experiences and current state of mind may influence the quality of the relationship with their child (Berthelot et al., 2015; Milot et al., 2014; Sauvé et al., 2021). Interventions should aim to strengthen this relationship and promote the development of sensitive parenting behaviors. Many researchers recommend using attachment‐based intervention programs such as STEEP (Steps Toward Effective, Enjoyable Parenting; Egeland & Erickson, 2004), the Parallel Parent and Child Therapy (PPACT; Chambers et al., 2006) or the Attachment Video‐feedback intervention (AVI; Dubois‐Comtois et al., 2017; Moss, et al., 2011), which have been shown to be effective among high‐risk populations.

Furthermore, results from this review support the need to invest in the development of instruments designed to capture HH indicators and screen for HH attachment representations (Finger et al., 2015). Although the AAI is an effective screening instrument, the costs associated with training (both in regard to administrating and coding) limit its use during or prior to psychotherapy. Additional measures such as short clinical interviews with adapted assessment guidelines are needed to support clinicians in identifying patterns of disorganized attachment representations. Clinical indicators of a HH state of mind can be difficult to identify and require careful attention on behalf of the clinician in order to identify inconsistencies and contradictions in the individual's discourse that may be unacknowledged or dismissed. As a note of caution, it is also important to mention that, although HH indicators can be used as a proxy to assess parenting abilities, researchers emphasize that the purpose of screening is not to inform child welfare services about whether or not children should be removed from their family, but rather to intervene in order to optimize the quality of parent‐child interactions (Terry et al., 2021).

### 4.3 Limitations

This paper is the first to systematically review and integrate findings related to HH states of mind. This scoping review makes significant contributions to our understanding of this form of adult attachment disorganization by providing a synthesis of research on HH states of mind as a consequence of childhood relational trauma, a risk factor for maladaptive psychological functioning and a potential mediating factor in the intergenerational transmission of maladaptation, while highlighting the unique contributions of the HH construct compared to other forms of attachment disorganization. Nonetheless, findings must be interpreted in light of certain limitations. First, although 19 studies were included in the synthesis, a number of these publications were conducted with the same sample at different points in time, resulting in the inclusion of data from only nine independent samples across articles. Second, the limited number of studies and the large scope of research questions examined in these studies resulted in the prioritization of a scoping review, which does not allow for statistical analyses. However, it is widely recognized that scoping reviews are a preliminary step to a systematic literature review and/or meta‐analysis (Arksey & O'Malley, 2005). Caution should also be exercised in generalizing the results as the review includes multiple study designs and methods, most papers were based on small sample sizes, and less than half of studies included a comparison group. Finally, the current literature on HH states of mind focuses primarily on past relational traumas and does not consider all types of traumas, such as those perpetuated by systems (e.g., discrimination, structural racism, implicit bias). It is important to examine the ways in which such traumatic experiences in adulthood may play a role in perpetuating the intergenerational cycle of trauma.

## 5 CONCLUSION

Interpersonal trauma is a critical social and public health issue that increases the risk of disorganized attachment which, in turn, is associated with psychosocial maladjustment. Findings from this scoping review support the predictive validity of the HH coding system in detecting adult disorganized attachment representations in clinical and high‐risk populations. Results suggest that HH states of mind are related to many dimensions of the family ecology and are implicated in the intergenerational transmission of maladaptation. They highlight the need to identify individuals who are at risk or already present HH characteristics to help them revise their mental representations. Future studies are needed to explore mechanisms potentially involved in the development of HH states of mind and the intergenerational transmission of maladaptation, as well as to document the effectiveness of interventions in reducing the severity of symptoms, revising and possibly reversing disorganized attachment representations in adulthood, and preventing the transmission of risk from one generation to the next.

## CONFLICT OF INTEREST STATEMENT

No potential conflict of interest was reported by the authors.

References marked with an asterisk are those included in the synthesis.

## Supporting information

Supporting Information

## Footnotes

1 The CC state of mind classification is used in cases where individuals present contradictory strategies but fail to meet the requirements of a single classification (Main et al., 2002). Due to the low coherence of these protocols, they are considered insecure (Hesse, 2016).

2 A person with a score of 5 or above is considered to have a Hostile‐Helpless state of mind.

3 For ease of reading, these participants are referred to as "young adults" in the remainder of the article.
