# Supplementary material for: Hostile‐helpless states of mind: A scoping review of risk factors, correlates, and consequences
Source: Infant Ment Health J. 2022 May 31;43(4):597–623. doi: 10.1002/imhj.21994 (PMC9542117; doi:10.1002/imhj.21994)
Supplement: Supplementary file 1 — Supporting Information [file IMHJ-43-597-s001.docx]

| **Table S1:** Quality assessment of studies included in the synthesis | | | | | | | | | | | | | | | | | | | | |  | | |
| --- | --- | --- | --- | --- | --- | --- | --- | --- | --- | --- | --- | --- | --- | --- | --- | --- | --- | --- | --- | --- | --- | --- | --- |
| Authors & year | Were the aims / objectives of the study clear? | Was the study design appropriate for the stated aim(s)? | Was the sample size justified? | Was the target/reference population clearly defined? | Was the sample frame taken from an appropriate population base so that it closely represented the target/reference population under investigation? | Was the selection process likely to select subjects/participants that were representative of the target/reference population under investigation? | Were measures undertaken to address and categorise non-responders? | Were the risk factor and outcome variables measured appropriate to the aims of the study? | Were the risk factor and outcome variables measured correctly using instruments/measurements that had been trialled, piloted or published previously? | Is it clear what was used to determined statistical significance and/or precision estimates? (e.g. p-values, confidence intervals) | Were the methods (including statistical methods) sufficiently described to enable them to be repeated? | Were the basic data adequately described? | Does the response rate raise concerns about non-response bias? | If appropriate, was information about non-responders described? | Were the results internally consistent? | Were the results presented for all the analyses described in the methods? | Were the authors' discussions and conclusions justified by the results? | Were the limitations of the study discussed? | Were there any funding sources or conflicts of interest that may affect the authors interpretation of the results? | Was ethical approval or consent of participants attained? | | Number of correct answers (score out of 20) |  |
| Barone et al. (2014) | Y | Y | N | Y | Y | Y | NM | Y | Y | Y | Y | Y | N | NM | Y | Y | Y | Y | N | Y | | 17 |  |
| Barone and Carone (2020) | Y | Y | N | Y | Y | Y | NM | Y | Y | Y | Y | Y | N | NM | Y | Y | Y | Y | N | Y | | 17 |  |
| Barone and Frigerio (2009) | Y | Y | N | Y | Y | Y | Y | Y | Y | Y | Y | Y | N | Y | Y | Y | Y | Y | N | Y | | 19 |  |
| Brumariu et al. (2013) | Y | Y | Y | Y | Y | Y | Y | Y | Y | Y | Y | Y | N | Y | Y | Y | Y | Y | N | Y | | 20 |  |
| Byun et al. (2016) | Y | Y | N | Y | Y | Y | Y | Y | Y | Y | Y | Y | N | Y | Y | Y | Y | Y | N | Y | | 19 |  |
| Finger (2006) | Y | Y | N | Y | Y | Y | NM | Y | Y | Y | Y | Y | N | NM | Y | Y | Y | Y | N | Y | | 17 |  |
| Finger et al. (2015) | Y | Y | N | Y | Y | Y | Y | Y | Y | Y | Y | Y | N | Y | Y | Y | Y | Y | N | Y | | 19 |  |
| Frigerio et al. (2013) | Y | Y | N | Y | Y | Y | NM | Y | Y | Y | Y | Y | N | NM | Y | Y | Y | Y | N | Y | | 17 |  |
| Guarino et al. (2011) | Y | Y | N | Y | Y | Y | NM^[[1]](#footnote-1)^ | Y | Y | Y | Y | Y | N | NM | Y | Y | Y | Y | N | Y | | 17 |  |
| Honde (2007) | Y | Y | N | Y | Y | Y | NM | Y | Y^[[2]](#footnote-2)^ | Y | Y | Y | N | NM | Y | Y | Y | Y | N | Y | | 17 |  |
| Lyons-Ruth et al. (2007) | Y | Y | N | Y | Y | Y | NM^3^ | Y | Y | Y | Y | Y | N | NM | Y | Y | Y | Y | N | Y | | 17 |  |
| Lyons-Ruth et al. (2003) | Y | Y | N | Y | Y | Y | Y | Y | Y | Y | Y | Y | N | Y | Y | Y | Y | Y | N | NM | | 18 |  |
| Lyons-Ruth et al. (2005) | Y | Y | N | Y | Y | Y | Y | Y | Y | Y | Y | Y | N | Y | Y | Y | Y | N | N | NM | | 17 |  |
| Milot et al. (2014) | Y | Y | N | Y | Y | Y | Y | Y | Y | Y | Y | Y | N^[[3]](#footnote-3)^ | Y | Y | Y | Y | Y | N | NM | | 18 |  |
| Obsuth et al. (2014) | Y | Y | N | Y | Y | Y | Y | Y | Y | Y | Y | Y | N | Y | Y | Y | Y | Y | N | NM | | 18 |  |
| Sauvé et al. (2021) | Y | Y | N | Y | Y | Y | NM | Y | Y | Y | Y | Y | N | NM | Y | Y | Y | Y | N | Y | | 17 |  |
| Terry et al. (2021) | Y | Y | N | Y | Y | Y | NM | Y | Y | Y | Y/N^[[4]](#footnote-4)^ | Y | N | NM | Y | Y | Y | Y | N | Y | | 16 |  |
| Vulliez‐Coady et al. (2013) | Y | Y | N | Y | Y | Y | Y | Y | Y | Y | Y | Y | N | Y | Y | Y | Y | Y | N | NM | | 18 |  |
| Yellin (2001) | Y | Y | N | Y | Y | Y | Y | Y | Y | Y | Y | Y | N | Y | Y | Y | Y | Y | N | NM | | 18 |  |

***Note.*** Y = yes, N = no; NM: not mentioned.

1. Information about excluded data/participants is presented. [↑](#footnote-ref-1)
2. Interrater reliability for HH states of mind was not calculated. [↑](#footnote-ref-2)
3. Mothers lost to attrition were not significantly different on any of the sociodemographic variables. [↑](#footnote-ref-3)
4. Yes for quantitative analysis, no for qualitative analysis. [↑](#footnote-ref-4)
